# Impact of surgery in patients with multiple sclerosis: a nationwide cohort study

**DOI:** 10.3389/fneur.2025.1573349

**Published:** 2025-06-26

**Authors:** Emma Larsson, Ellen Iacobaeus, Erik von Oelreich, Jesper Eriksson, Jessica Kåhlin

**Affiliations:** ^1^Department of Physiology and Pharmacology, Karolinska Institutet, Stockholm, Sweden; ^2^Department of Perioperative Medicine and Intensive Care, Karolinska University Hospital Solna, Stockholm, Sweden; ^3^Department of Clinical Neuroscience, Karolinska Institutet, Stockholm, Sweden; ^4^Department of Neurology, Karolinska University Hospital, Stockholm, Sweden

**Keywords:** multiple sclerosis, surgery, perioperative care, health care utilization, postoperative outcome

## Abstract

**Background:**

Surgery is a common exposure. Multiple sclerosis (MS) is a chronic neuroinflammatory demyelinating disease of the central nervous system and a systemic inflammatory activation caused by surgery may result in exacerbation of the disease. It is unknown how surgical procedures affect morbidity and mortality rates in MS.

**Objectives:**

This study aimed to investigate morbidity associated with surgical interventions in MS patients by assessing disease burden before and after surgery. Non-MS patients were used as controls, allowing for comparisons of disease burden and mortality between the two groups.

**Methods:**

The cohort study analyzed data from the Swedish Perioperative Register, including 3,022 MS patients among over 1.5 million surgeries performed between January 2019 and March 2023. Disease burden was measured as the number of pre-specified ICD-codes before and after surgery.

**Results:**

We demonstrated that MS patients exhibited a higher mean number of diagnoses before and after surgery compared to controls. Specifically, the number of diagnoses peaked in the first month post-surgery but returned to baseline within three to 4 months. Notably, there were no significant differences in 30-day or 365-day mortality rates between MS and non-MS patients, highlighting the relative safety of surgical interventions for persons with MS.

**Conclusion:**

The findings suggest that surgery is generally safe for patients with MS, indicating that MS should not preclude necessary surgical interventions. Nevertheless, tailored preoperative assessments and postoperative care strategies are essential to address the unique health challenges encountered by MS patients, ensuring optimal surgical outcomes and monitoring for potential complications.

## Introduction

One in ten individuals in high income countries undergo surgery each year (1), exposing large volumes of people worldwide to a sterile trauma with short and long-term effects on the immune system as well as morbidity.

Multiple sclerosis (MS) is a chronic neuroinflammatory autoimmune disease that affects the central nervous system. MS is one of the most frequent causes of neurological disability among young adults and has a worldwide prevalence of around 2.8 million (2). Clinical onset of the disease is usually between the ages of 20 and 40 years but the subsequent evolution of disease activity and long-term outcome is highly variable between individuals (3).

Patients with MS suffer an increased risk for several comorbidities such as other autoimmune diseases, cardiovascular disease and depression (4). Growing evidence has pointed out comorbidity as an important prognostic factor for disease outcome in MS (5).

Comorbidity has been suggested to associate with MS disease activity (6) and several comorbidities increase the risk of MS disease progression (7).

The risk of intensive care unit (ICU) admission is significantly higher in individuals with MS compared with the general population, with a notably elevated 1-year mortality rate following such admissions (8). However, in the context of surgery, it remains unclear how the associated trauma might influence MS symptoms, morbidity, or the overall clinical progression of the disease. A meta-analysis of bariatric surgery outcomes in patients with MS concluded that there was no significant increase in surgery-related or overall postoperative complications within the MS cohort (9). Recent data also suggest that the risk of a defined or typical MS relapse after surgery is not elevated (10), although earlier reports present conflicting findings (11, 12). This discrepancy underscores the importance of studying the effects of surgery in MS patients, as surgery is a common medical intervention and to understand how surgery impacts on MS disease activity is critical to improve clinical care. Moreover, determining whether surgery exacerbates MS symptoms or leads to relapses can guide clinicians in making informed decisions on surgical risk assessments and interventions.

Studies elucidating disease burden before and after surgery in patients with MS are lacking and could provide valuable knowledge on disease trajectory under inflammatory stress.

In this study, we aimed to assess disease burden associated with surgery by analyzing the number of pre-specified ICD-codes before and after surgery in MS patients undergoing surgery in Sweden between January 1, 2019, and March 21, 2023. We also investigated health care utilization/hospital length of stay after acute and elective surgery. Non-MS patients were used as controls, allowing for comparisons of disease burden and mortality between the two groups. This analysis provides valuable insights regarding morbidity and mortality associated with surgery in MS patients and provides practical implications for the care of MS patients undergoing surgery.

## Materials and methods

### Setting

This multicenter cohort study utilized prospectively collected data on virtually all surgical procedures performed in Sweden on adult patients with a diagnosis of MS between January 1, 2019, and March 21, 2023. The Swedish personal identity number is assigned to every resident, facilitating extensive register linkages (13). Sweden maintains a healthcare system defined by tax-funded and universally accessible services. The study was approved by the Swedish Ethical Review Authority (Approval Number 2021-02906), which waived the need for informed consent from participants. The study adhered to the STROBE (Strengthening the Reporting of Observational Studies in Epidemiology) guidelines for cohort studies (14). All research was conducted in accordance with national guidelines and regulations.

### Registers and study population

The Swedish Perioperative Register (SPOR) has a national coverage of approximately 98% and contains detailed surgical data. Surgical procedures are classified into subtypes based on surgical codes used in the Nordic countries (NOMESCO). SPOR holds data on type of surgery, diagnoses, The American Society of Anesthesiologists Physical Status (ASA-PS) (15) and quality measures. Data are automatically transferred electronically from the medical journal systems to SPOR, with a recent publication which revealed good agreement between local and central databases (16). Written informed consent is not required, but patients may withdraw their data from the register at any time.

In this nationwide cohort study, we identified all surgical procedures performed on patients 18 years or older, from January 1, 2019, to March 21, 2023, in SPOR, except for surgeries involving dermatological procedures, procedures classified as “minor surgical procedures,” transluminal endoscopic procedures, diagnostic procedures in connection with other surgical procedures, organ donor surgeries and procedures categorized under “additional” codes.

**Figure 1:**
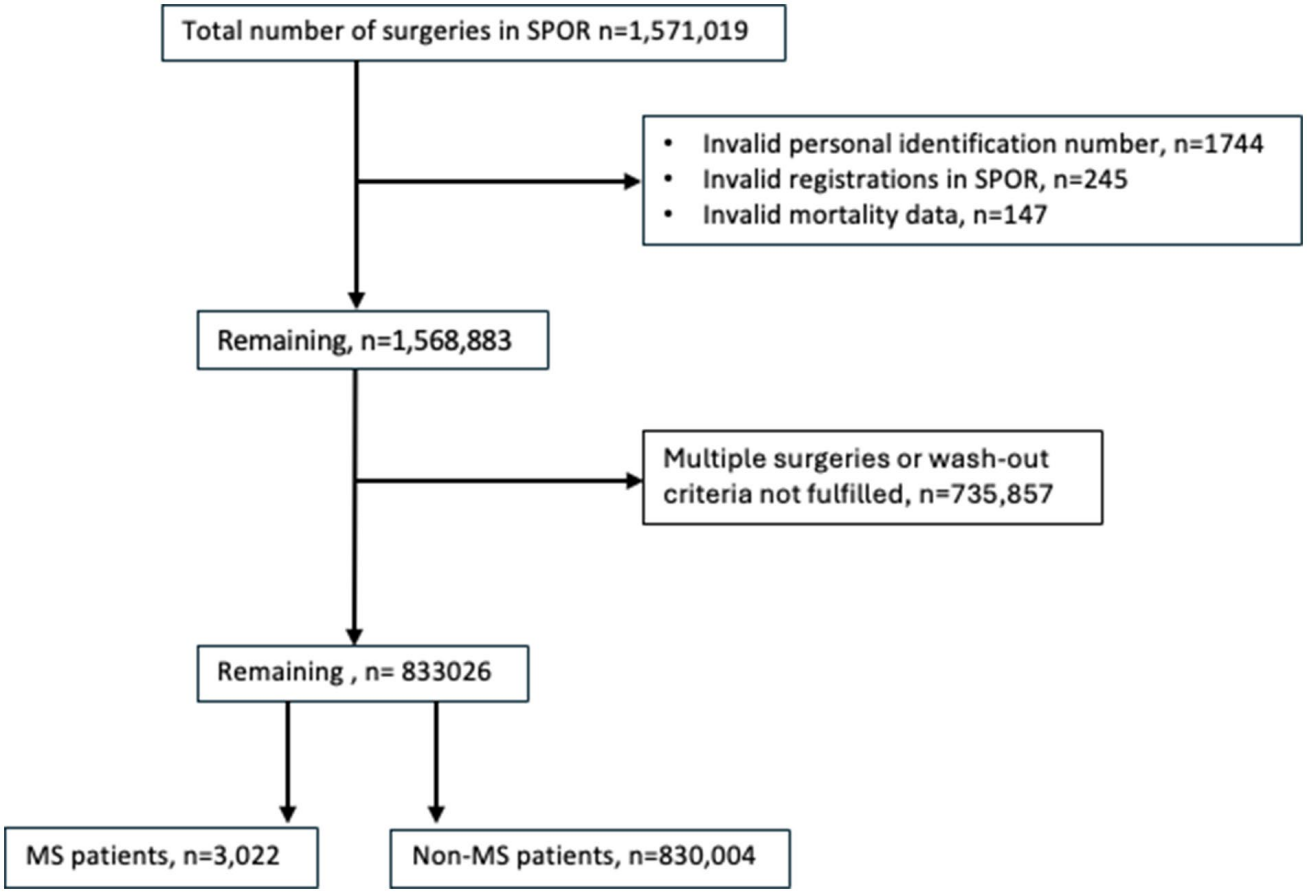
Flowchart of included patients.

We excluded surgical procedures performed in patients with temporary or invalid personal identification numbers, invalid registrations in SPOR and patients with invalid mortality data. Surgical procedures performed between January and June 2019 were excluded to allow for a wash-out period for any re-operations prior to the index surgery. Similarly, to avoid including re-operations following the index surgery, a surgery-free interval of at least 6 months after the index procedure was required. If multiple surgeries met these criteria, the first qualifying procedure was selected (Figure 1). Patients with MS were defined as having a diagnosis of MS (ICD-10 code G35.9) in the Swedish National Patient Register before their respective index surgery (17).

The selection of diagnoses assessed before and after surgery were based on previous findings describing the most prevalent conditions in MS populations (18). We also included diagnoses for commonly reported symptoms in MS-patients including fatigue, psychiatric conditions (anxiety and depression), symptoms related to bladder—and gastrointestinal disturbance, pain, in addition to infection. The International Classification of Diseases (ICD) codes for the comorbidities were used to retrieve data from the National Patient Register. A comprehensive list of the included ICD codes can be found in Supplementary Table 1. These codes were grouped into distinct categories, including fatigue, urology, pain, infection, psychiatric conditions, gastrointestinal issues, and others. We collected ICD codes for analysis covering the 12 months prior to and the 12 months following the surgical procedures. Moreover, the National Patient Register provided data on index hospital admission and hospital discharge dates, re-admission and subsequent discharge dates.

Data on education and income were retrieved from Statistics Sweden (19). Education level at the time of the surgical procedure was categorized as low, medium, or high, corresponding to 9 years or less (primary school), 10–12 years (secondary school) and more than 12 years (university level), respectively. Income in the year before surgery was classified into low, medium, and high corresponding to less than half of the median national income, between half to double the median national income and more than double the median national income, respectively. The Cause of Death Register provided mortality data (20).

### Outcomes and covariates

The primary outcome was the monthly frequency of pre-specified ICD-coded diagnoses in patients with multiple sclerosis (MS) undergoing surgery, based on the diagnostic codes listed in Supplementary Table 1. Diagnoses were recorded monthly for the 12 months preceding and up to 12 months following the surgical procedure. The mean number of diagnoses per month in MS patients was compared to that of non-MS patients. In addition, we conducted separate analyses for MS patients to assess when their comorbidity burden, measured by the monthly frequency of ICD-coded diagnoses, returned to baseline, defined as the average number of diagnoses during months 7 to 12 prior to surgery.

Secondary outcomes included Days Alive and at Home within 30 days (DAH30), calculated according to the method described by Myles et al. (21). DAH30 is derived from the date of index surgery (Day 0) using hospitalization and mortality data, with length of hospital stay determined from the surgery and discharge dates. We also calculated DAH90 using the same approach. Additional secondary outcomes included the proportion of days spent in hospital for each month before and after surgery, as well as crude 30-day and 365-day mortality rates.

### Statistical analyses

Categorical variables are presented as counts with percentages, while continuous variables are reported as means with standard deviations (SD) and medians with interquartile ranges (IQR). Comparisons of proportions were performed using the chi-square test. Continuous variables were compared using either the Student’s *t*-test or the Mann–Whitney *U* test, as appropriate.

Generalized estimating equations (GEE) regression models were utilized to estimate differences in mean number of MS-related diagnoses between MS patients and non-MS patients across 12 months before and 12 months after surgery. The GEE model was adjusted for age, sex, income level, and educational attainment. Comorbidity and the American Society of Anesthesiologists (ASA) physical status classification were not included in the adjustments, as these measures may capture downstream consequences of multiple sclerosis, such as neurological impairment or reduced functional capacity. Including them in the model could result in overadjustment and potentially obscure the true association between MS and postoperative outcomes.

To assess when the comorbidity burden in MS patients, measured by the monthly frequency of ICD-coded diagnoses, returned to baseline, we used GEE without covariate adjustment. As each patient was compared to their own preoperative baseline, this within-person comparison inherently controlled for time-invariant individual characteristics, including demographic factors.

GEE analyses were performed using robust variance estimators and an independent correlation structure. A *p*-value of less than 0.05 was considered statistically significant, with all tests being two-tailed. Data analysis was performed using Stata/MP 16.1 (StataCorp, College Station, TX).

### Missing data

The study included a low rate of missing data. In the final patient cohort, the only variables with missingness were ASA-score (missing data in 10.0% of the cases), education level (2.0% missing) and income level (0.3% missing). No major differences were seen between patients with and without missing data on ASA-score except for a higher frequency of thoracic surgery in patients with missing data on ASA-score (data not shown). For the variables included in the analyses, the proportion of missing data for education and income was below 5%, a threshold commonly considered acceptable for complete-case analysis in large datasets (22). No other variables used in the analyses had missing data. Therefore, all analyses were conducted using a complete-case approach.

## Results

The study population was derived from the SPOR, comprising a total of 1,571,019 recorded surgeries. Surgeries with invalid personal identity numbers and registrations deemed invalid in SPOR (*n* = 1,989), and those with invalid mortality data (*n* = 147) were excluded. Additionally, multiple surgeries or those not meeting wash-out criteria were excluded (*n* = 735,857). Following these exclusions, the final study cohort consisted of 3,022 patients diagnosed with MS and 830,004 non-MS patients (Figure 1).

Patient and surgical procedure characteristics are presented in Table 1. The median age was 56 (43–68) years for MS patients and 57 (38–73) years for non-MS patients, with a higher proportion of women among the MS cohort. The differences in comorbidity, as measured by the Charlson Comorbidity Index (CCI), were approximately equal; however, MS patients exhibited a higher ASA classification compared to non-MS patients. The most common surgical procedure was orthopedic surgery, followed by abdominal surgery, in both groups. Approximately one-third of the procedures were classified as acute for both groups.

**Table 1:** Patient and surgical procedure characteristics.

Patient and surgical procedure characteristics	Non-MS	MS	*p*-value
	*N* = 830,004	*N* = 3,022	
Age, median (IQR)	57 (38–73)	56 (43–68)	0.088
Male, count *n* (%)	339,699 (40.9%)	772 (25.5%)	<0.001
Income level, count *n* (%)			<0.001
Low	149,231 (18.0%)	503 (16.6%)	
Medium	619,788 (74.9%)	2,348 (77.7%)	
High	58,254 (7.0%)	171 (5.7%)	
Education level, count *n* (%)			<0.001
Low	166,168 (20.4%)	430 (14.3%)	
Medium	363,401 (44.7%)	1,378 (45.9%)	
High	284,108 (34.9%)	1,194 (39.8%)	
CCI categories, count *n* (%)			0.050
CCI 0	554,430 (66.8%)	1,959 (64.8%)	
CCI 1	91,938 (11.1%)	342 (11.3%)	
CCI ≥2	183,636 (22.1%)	721 (23.9%)	
ASA score, median (IQR)			<0.001
ASA score 1	215,441 (28.8%)	124 (4.5%)	
ASA score 2	360,028 (48.2%)	1,428 (52.1%)	
ASA score 3	155,257 (20.8%)	1,083 (39.5%)	
ASA score ≥4	16,210 (2.2%)	105 (3.8%)	
Comorbidities			
Acute myocardial infarction	32,103 (3.9%)	98 (3.2%)	0.075
Congestive heart failure	35,212 (4.2%)	97 (3.2%)	0.005
Peripheral vascular disease	22,341 (2.7%)	85 (2.8%)	0.68
Cerebrovascular disease	37,257 (4.5%)	147 (4.9%)	0.32
Dementia	10,770 (1.3%)	28 (0.9%)	0.072
COPD	46,717 (5.6%)	174 (5.8%)	0.76
Rheumatic disease	20,317 (2.4%)	78 (2.6%)	0.64
Peptic ulcer disease	8,909 (1.1%)	30 (1.0%)	0.67
Mild, moderate, or severe liver disease	11,523 (1.4%)	32 (1.1%)	0.12
Diabetes with or without complications	63,982 (7.7%)	220 (7.3%)	0.38
Hemiplegia or paraplegia	8,775 (1.1%)	187 (6.2%)	<0.001
Renal disease	20,351 (2.5%)	44 (1.5%)	<0.001
Cancer with or without metastasis	112,043 (13.5%)	351 (11.6%)	0.002
Alcohol or substance abuse	24,015 (2.9%)	72 (2.4%)	0.094
Depression or psychoses	47,787 (5.8%)	239 (7.9%)	<0.001
Hospital size, count *n* (%)			<0.001
County hospital	265,171 (31.9%)	844 (27.9%)	
Central hospital	313,879 (37.8%)	1,144 (37.9%)	
University hospital	250,954 (30.2%)	1,034 (34.2%)	
Type of surgery, count *n* (%)			<0.001
Neuro surgery	43,243 (5.2%)	280 (9.3%)	
Endocrine & breast surgery	40,386 (4.9%)	175 (5.8%)	
ENT surgery	40,471 (4.9%)	121 (4.0%)	
Pulmonary & thoracic surgery	39,543 (4.8%)	97 (3.2%)	
Abdominal surgery	177,529 (21.4%)	608 (20.1%)	
Urological surgery	59,374 (7.2%)	267 (8.8%)	
Gynecological surgery	75,881 (9.1%)	355 (11.7%)	
Obstetric surgery	77,953 (9.4%)	204 (6.8%)	
Orthopedic surgery	264,832 (31.9%)	885 (29.3%)	
Vascular surgery	10,792 (1.3%)	30 (1.0%)	
Acute surgery	290,369 (35.0%)	1,012 (33.5%)	0.085

MS, multiple sclerosis.

**Figure 2:**
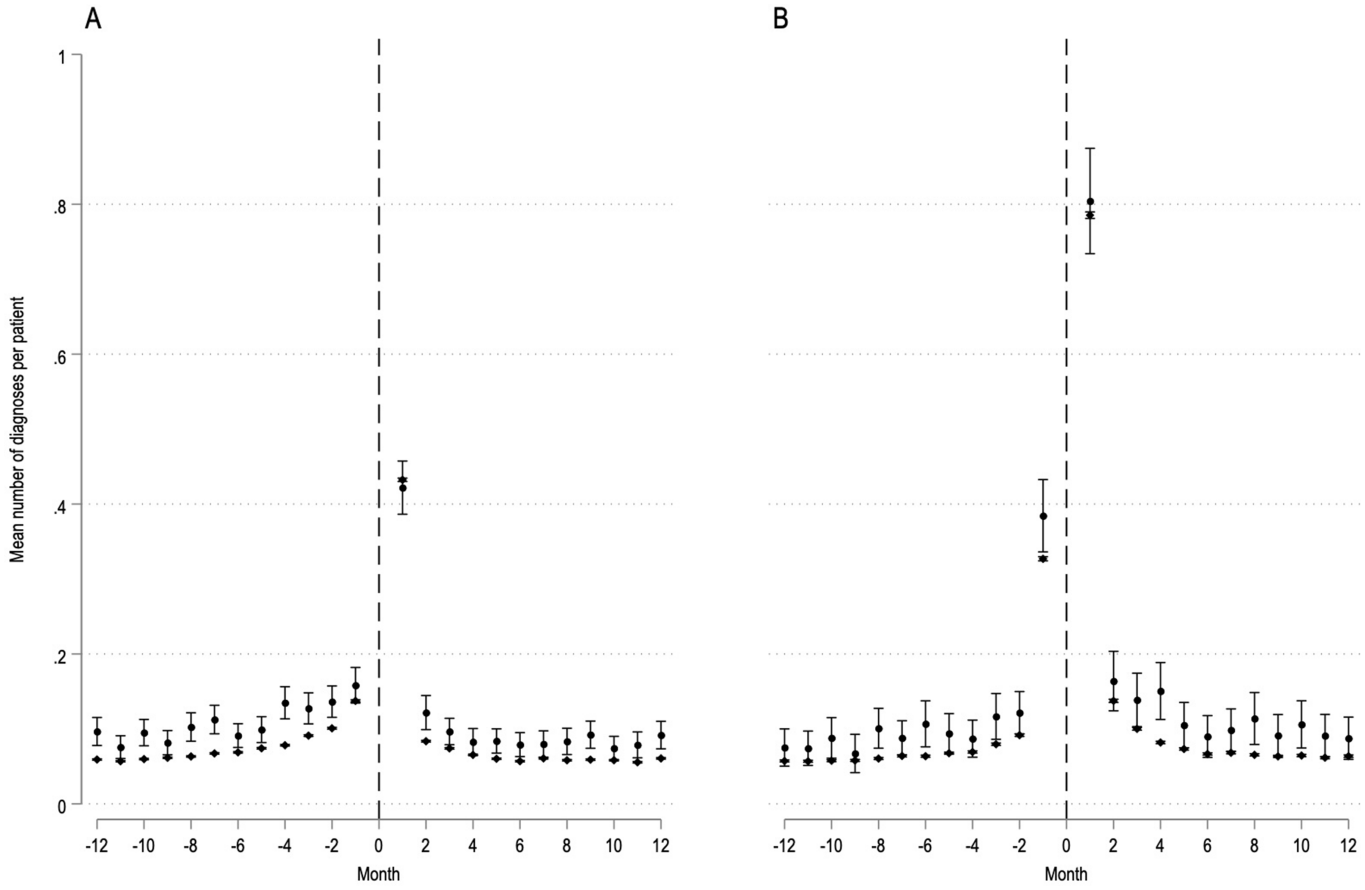
MS-related diagnoses before and after elective (panel A) and acute (panel B) surgery. Patients with MS (n = 3,022) are depicted with circles, patients without MS (n = 830,004) are depicted with diamonds. Dashed line represents time of surgery. Y-axis, mean number of MS-related diagnoses before and after surgery per patient with 95% confidence intervals. X-axis, month before and after surgery.

In elective surgery, the mean number of comorbidities was significantly higher in MS patients compared with non-MS patients across all months, both before and after surgery, except the month immediately after surgery. Similar results were noted after adjustments (Figure 2 and Supplementary Table 2). The mean number of comorbidities in MS patients began to increase around 4 months before surgery and peaked in the first month after surgery which was followed by a steep decline and return to baseline by the third month after surgery. Baseline was defined as the mean number of diagnoses 7–12 months before surgery (Supplementary Table 3).

In acute surgery, the mean number of comorbidities was generally higher in MS patients compared to non-MS patients both before and after surgery; however, the differences did not reach statistical significance for each individual month. Similar results were noted after adjustments (Figure 2 and Supplementary Table 4). The mean number of comorbidities showed a similar pattern before and after surgery as observed in elective surgery. Specifically, comorbidities began to rise significantly 3 months before surgery. This trend peaked during the first month after surgery and returned to baseline by the fourth month (Supplementary Table 5).

There was no statistically significant unadjusted difference in 30-day or 365-day mortality between MS and non-MS patients, regardless of whether the surgical procedures were elective or acute. MS patients exhibited slightly lower unadjusted DAH30 and DAH90 after both elective and acute surgery (Table 2).

**Table 2:** Mortality and Days Alive and at Home (DAH).

Mortality and DAH	Non-MS	MS	*p*-value
Elective surgery	*n* = 539,635	*n* = 2,010	
Mortality, count *n* (%)			
30-day	1,224 (0.2%)	6 (0.3%)	0.50
365-day	10,460 (1.9%)	46 (2.3%)	0.26
DAH			
DAH30, mean (SD)	28.4 (3.6)	27.8 (4.6)	<0.001
DAH30, median (IQR)	30 (28–30)	29 (28–30)	<0.001
DAH90, mean (SD)	87.6 (8.2)	86.8 (9.6)	<0.001
DAH90, median (IQR)	90 (88–90)	89 (88–90)	<0.001
Acute surgery	*n* = 290,369	*n* = 1,012	
Mortality, count *n* (%)			
30-day	9,606 (3.3%)	23 (2.3%)	0.066
365-day	25,142 (8.7%)	82 (8.1%)	0.53
DAH			
DAH30, mean (SD)	24.8 (7.5)	23.9 (7.5)	<0.001
DAH30, median (IQR)	28 (24–29)	27 (22–29)	<0.001
DAH90, mean (SD)	80.6 (20.9)	79.9 (19.7)	0.26
DAH90, median (IQR)	88 (84–89)	86 (81–89)	<0.001

MS, multiple sclerosis.

**Figure 3:**
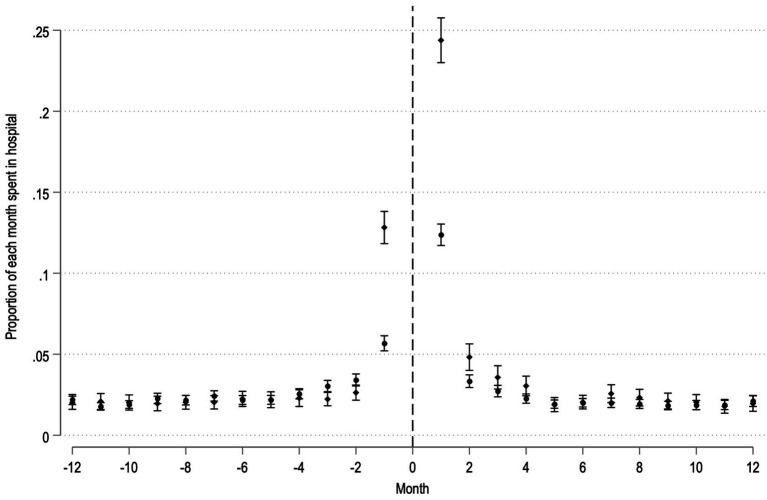
Proportion of each month spent in hospital for MS-patients before and after elective (circles) and acute (diamonds) surgery. Dashed line represents time of surgery. Y-axis, Proportion of respective month spent admitted to hospital with 95% confidence intervals. X-axis, month before and after surgery.

When analyzing the proportion of each month that MS-patients were admitted to the hospital before and after surgery, MS-patients proportion of monthly admittance seemed to return to baseline after a few months, regardless of acute or elective surgery (Figure 3).

## Discussion

### Key findings

In this multicenter cohort study, we investigated the surgical outcomes and comorbidity profiles of patients with MS in comparison to non-MS patients. Our findings indicate that while MS patients had a higher mean number of comorbidities both before and after surgery, there were no significant differences in 30-day or 365-day mortality rates between the two groups. Additionally, MS patients experienced an increase in comorbidities in the months leading up to surgery; however, these levels returned to baseline within a few months after surgery. MS patients undergoing both elective and acute surgery had a slightly lower DAH30 and DAH90, reflecting a marginally extended course of care postoperatively. Finally, after surgery in MS-patients, health care utilization returns to baseline a few months postoperatively both for elective and acute surgery. In summary, our results suggest that a diagnosis of MS should not preclude patients from undergoing surgical procedures when indicated. However, it is crucial to emphasize the importance of tailored preoperative assessments and postoperative care strategies and clinical follow up for MS patients to identify and treat possible occurrence of comorbidities.

### Previous studies

Prior studies exploring possible negative effects of surgery, including such as onset of a new relapse, new symptoms/disorders and disability progression in MS are sparse. A previous study assessed 69 surgeries with anesthesia in 281 MS patients and found no increase in relapse risk during a postoperative period of 90 days (10, 23). Furthermore, no association between risk of MS and exposure to anesthesia was observed in a population-based Swedish study where neither general anesthesia, nor regional techniques were found to induce MS relapses (23, 24). However it is in general advised to use neuromuscular blocking agents (NMBA) with caution due to risk of upregulation of their targets, acetylcholine receptors, with following excessive potassium release or resistance to NMBA, depending on the characteristic of the drug (25). A prior systematic meta-analysis found an increased risk for developing MS in patients that underwent tonsillectomy and appendectomy at an age of 20 years or younger but there was no evidence of an association between other surgeries and the risk for MS (26).

Surgery induces a sterile inflammatory surge of damage associated molecular patterns (DAMPs) from damaged cells upon incision, propagated through cytokine release from leukocytes and subsequent activation of innate and adaptive immune responses (27, 28). The systemic inflammation induced by surgery is suggested to result in imprints on the brain with resulting neuroinflammation triggering cognitive decline and potentially increased risk for later development of dementia (29, 30). However, there is currently no consensus regarding the risk of surgery-induced inflammation to result in disability worsening or relapse activity in MS. Our findings suggest that MS patients return to a frequency of comorbidities and healthcare visits comparable to baseline levels within three to 4 months post-surgery. Smaller studies exploring the impact of surgery on the clinical course of MS patients have yielded similar results. A systematic review by Shasavan et al. (9) concluded that the complication rates in 394 patients with MS undergoing bariatric surgery were equal to patients without MS. The included studies all had a limited number of patients but changes in disease activity and outcome had been assessed with clinical tools and questionnaires. To the best of our knowledge the current study is the largest study examining surgical outcome in MS patients. In this population-based study, we investigated the increased comorbidity burden that may arise from the inflammatory peak induced by a surgical intervention. Rather than relying on individual evaluation tools, our outcomes are based on population data, utilizing a larger cohort than in previous studies.

The main reason for including only the first surgery and a defined wash-out period in this study was to maintain the internal validity of our findings. Our aim was to specifically explore disease burden in MS patients in relation to a clearly defined surgical event. Including re-operations or surgeries that occurred in close proximity could introduce ambiguity, making it difficult to discern whether any observed changes in health status were attributable to the surgery under investigation or to earlier interventions.

### Strengths and limitations

The use of nationwide registries provides a large and representative sample of the population, enhancing the generalizability of the findings to the broader population of patients with MS. The Swedish national health registries cover a wide range of healthcare data, including diagnoses, surgical procedures, hospital admissions, and mortality, allowing for a comprehensive investigation of comorbidities and surgical outcomes. Data is prospectively reported to SPOR for quality-surveillance purposes and therefore unbiased in relation to this project. However, we recognize several limitations that must be addressed.

Firstly, while the registry data is extensive, there remains a potential for misclassification of MS or comorbidities due to inconsistencies or inaccuracies in coding and data entry. This may affect the validity of some of our conclusions. Secondly, due to the registry-based nature of the study, we lack detailed clinical information such as disease severity, disease activity, and exact clinical indications for surgery. This limits the ability to fully understand the impact of MS-related factors on surgical outcomes. Additionally, we were unable to distinguish between MS subtypes, which could exhibit varying baseline comorbidities and postoperative risks, potentially affecting the study’s findings.

The absence of data on neurological disability impairment and immunomodulatory treatments further limits the depth of our analysis. It is also important to consider that the study’s inclusion period overlapped with the COVID-19 pandemic, a time of significant strain on the healthcare system, which could have influenced both the availability and quality of care during the study period.

Finally, while the data are derived from a Swedish cohort, the findings may not be directly generalizable to populations outside of Sweden due to differences in healthcare systems, population characteristics, and access to care.

## Conclusion—implications to study findings

Our results indicate that both elective and acute surgeries are generally safe for patients with MS. The findings reflect that, when clinically warranted, surgical interventions should proceed without unnecessary delay in MS patients, while acknowledging the importance of individualized risk evaluation. Furthermore, despite the overall safety of surgical interventions in MS patients, clinical vigilance is essential to monitor for any potential postoperative complications, occurrence of a new condition or symptom, or exacerbations/worsening of MS, or existing comorbidities. Careful preoperative assessments with evaluation of autonomic nervous system function and respiratory assessment as well as tailored postoperative care strategies including specific postoperative monitoring protocols to avoid venous thrombosis and postoperative infection can help address the unique health challenges faced by this population, ensuring optimal outcomes.

Future studies on perioperative neurocognitive disorders (PND) after surgery in MS patients alongside high-resolution postoperative temporal systemic inflammatory characterization are of particular interest.

## Data Availability

The raw data supporting the conclusions of this article will be made available by the authors, without undue reservation.
