# Supplementary material for: Impact of surgery in patients with multiple sclerosis: a nationwide cohort study
Source: Front Neurol. 2025 Jun 26;16:1573349. doi: 10.3389/fneur.2025.1573349 (PMC12240756; doi:10.3389/fneur.2025.1573349)
Supplement: Supplementary file 1 [file Table_1.docx]

**Supplementary Table 1. Included ICD codes and defined sub-categories**

Wildcard; “?” denotes a single character of any kind. I.e “J01?” includes J010 – J019.

| **Sub-category** | **ICD code** |  | **Sub-category** | **ICD code** |  | **Sub-category** | **ICD code** |
| --- | --- | --- | --- | --- | --- | --- | --- |
|  |  |  |  |  |  |  |  |
| ***Other*** |  |  | ***Infection*** |  |  | ***Gastrointestinal*** |  |
| Sleep disorder, unspecified | G479 |  | Acute sinusitis | J01? |  | Gastro-oesophageal reflux disease | K21? |
| Disorders of initiating and maintaining sleep | G470 |  | Acute pharyngitis | J02? |  | Gastric ulcer | K25? |
| Postviral fatigue syndrome | G933 |  | Acute tonsillitis | J03? |  | Duodenal ulcer | K26? |
| Dysphagia | R13? |  | Acute laryngitis and tracheitis | J04? |  | Peptic ulcer, site unspecified | K27? |
| Paraesthesia of skin | R202 |  | Acute obstructive laryngitis [croup] and epiglottitis | J05? |  | Gastritis and duodenitis | K29? |
| Abnormalities of gait and mobility | R26? |  | Acute upper respiratory infections of multiple and unspecified sites | J06? |  | Other specified noninfective gastroenteritis and colitis | K528 |
| Other symptoms and signs involving cognitive functions and awareness | R41? |  | Pneumonia due to Streptococcus pneumoniae | J13? |  | Noninfective gastroenteritis and colitis, unspecified | K529 |
| Dizziness and giddiness | R42? |  | Pneumonia due to Haemophilus influenzae | J14? |  | Irritable bowel syndrome | K58? |
| Other specified fever | R508 |  | Bacterial pneumonia, not elsewhere classified | J15? |  | Other functional intestinal disorders | K590 |
| Fever, unspecified | R509 |  | Pneumonia due to other infectious organisms, not elsewhere classified | J16? |  | Functional diarrhoea | K591 |
| Malaise and fatigue | R53? |  | Pneumonia in diseases classified elsewhere | J17? |  | Cholelithiasis | K80? |
| Heat exhaustion, unspecified | T675 |  | Pneumonia, organism unspecified | J18? |  | Cholecystitis | K81? |
| Heat fatigue, transient | T676 |  | Acute bronchitis | J20? |  | Other diseases of gallbladder | K82? |
| Limitation of activities due to disability | Z736 |  | Acute bronchiolitis | J21? |  | Other specified diseases of biliary tract | K838 |
|  |  |  | Unspecified acute lower respiratory infection | J22? |  | Acute abdomen | R100 |
|  |  |  | Abscess of lung and mediastinum | J85? |  | Pain localized to upper abdomen | R101 |
|  |  |  | Pyothorax | J86? |  | Pain localized to other parts of lower abdomen | R103 |
|  |  |  | Postprocedural respiratory disorders, not elsewhere classified | J95? |  | Other and unspecified abdominal pain | R104 |
|  |  |  | Respiratory failure, not elsewhere classified | J96? |  | Dysphagia | R139 |
|  |  |  |  |  |  | Other specified symptoms and signs involving the digestive system and abdomen | R198 |
|  |  |  |  |  |  |  |  |
| ***Urology including urologic infections*** |  |  | ***Psychiatric*** |  |  | ***Cardiovascular diseases*** |  |
| Acute tubulo-interstitial nephritis | N109 |  | Schizophrenia | F20? |  | Pure hypercholesterolaemia | E780 |
| Acute cystitis | N300 |  | Schizoaffective disorders | F25? |  | Pure hyperglyceridaemia | E781 |
| Cystitis, unspecified | N309 |  | Bipolar affective disorder | F31? |  | Mixed hyperlipidaemia | E782 |
| Neuromuscular dysfunction of bladder, unspecified | N319 |  | Depressive Episode | F32? |  | Other hyperlipidaemia | E784 |
| Urinary tract infection, site not specified | N390 |  | Recurrent depressive disorder | F33? |  | Hyperlipidaemia, unspecified | E785 |
| Other specified urinary incontinence | N394 |  | Persistent mood [affective] disorders | F34? |  | Other disorders of lipoprotein metabolism | E788 |
| Other postprocedural disorders of genitourinary system | N998 |  | Phobic anxiety disorders | F40? |  | Disorder of lipoprotein metabolism, unspecified | E789 |
| Postprocedural disorder of genitourinary system, unspecified | N999 |  | Other anxiety disorders | F41? |  | Essential (primary) hypertension | I10? |
| Retention of urine | R339 |  |  |  |  | Angina pectoris | I20? |
| Other difficulties with micturition | R391 |  |  |  |  | Acute myocardial infarction | I21? |
| Observation for other suspected diseases and conditions | Z038 |  |  |  |  | Other forms of acute ischaemic heart disease | I248 |
|  |  |  |  |  |  | Acute ischaemic heart disease, unspecified | I249 |
|  |  |  |  |  |  | Chronic ischaemic heart disease, unspecified | I259 |
|  |  |  |  |  |  | Pulmonary embolism | I26? |
|  |  |  |  |  |  | Cardiomyopathy, unspecified | I429 |
|  |  |  |  |  |  | Other cardiomyopathies | I428 |
| ***Pain including migraine*** |  |  | ***Pulmonary diseases*** |  |  | Dilated cardiomyopathy | I420 |
| Migraine | G43? |  | Bronchitis, not specified as acute or chronic | J40? |  | Supraventricular tachycardia | I471 |
| Tension-type headache | G442 |  | Simple and mucopurulent chronic bronchitis | J41? |  | Ventricular tachycardia | I472 |
| Lumbago with sciatica | M544 |  | Unspecified chronic bronchitis | J42? |  | Paroxysmal tachycardia, unspecified | I479 |
| Dorsalgia, unspecified | M549 |  | Emphysema | J43? |  | Atrial fibrillation and flutter | I48? |
| Myalgia | M791 |  | Other chronic obstructive pulmonary disease | J44? |  | Heart failure | I50? |
| Neuralgia and neuritis, unspecified | M792 |  | Asthma | J45? |  | Cardiovascular disease, unspecified | I516 |
| Other chronic pain | R522 |  | Status asthmaticus | J46? |  | Phlebitis and thrombophlebitis | I80 |
| Pain, unspecified | R529 |  |  |  |  | Other venous embolism and thrombosis | I82 |
|  |  |  |  |  |  | Abnormal results of cardiovascular function studies | R943 |
|  |  |  |  |  |  |  |  |
|  |  |  |  |  |  |  |  |
|  |  |  |  |  |  |  |  |
|  |  |  |  |  |  |  |  |
|  |  |  |  |  |  |  |  |
|  |  |  |  |  |  |  |  |
|  |  |  |  |  |  |  |  |
