# Supplementary material for: Impact of surgery in patients with multiple sclerosis: a nationwide cohort study
Source: Front Neurol. 2025 Jun 26;16:1573349. doi: 10.3389/fneur.2025.1573349 (PMC12240756; doi:10.3389/fneur.2025.1573349)
Supplement: Supplementary file 2 [file Table_2.docx]

**Supplementary Table 2.** **Mean difference in number of MS-related diagnoses for patients with MS compared with patients without MS, before and after elective surgery*.**

| **Month before/after surgery** | **Mean difference (95% CI) in number of MS-related diagnoses** | **P value** |
| --- | --- | --- |
| -12 | 0.041 (0.023–0.059) | 0.000 |
| -11 | 0.025 (0.010–0.041) | 0.001 |
| -10 | 0.040 (0.023–0.058) | 0.000 |
| -9 | 0.025 (0.009–0.041) | 0.002 |
| -8 | 0.046 (0.027–0.065) | 0.000 |
| -7 | 0.050 (0.031–0.069) | 0.000 |
| -6 | 0.029 (0.013–0.045) | 0.000 |
| -5 | 0.031 (0.014–0.049) | 0.000 |
| -4 | 0.063 (0.042–0.085) | 0.000 |
| -3 | 0.041 (0.021–0.062) | 0.000 |
| -2 | 0.042 (0.021–0.063) | 0.000 |
| -1 | 0.027 (0.003-0.050) | 0.027 |
| 1 | -0.006 (-0.042-0.029) | 0.720 |
| 2 | 0.043 (0.021–0.066) | 0.000 |
| 3 | 0.028 (0.010–0.045) | 0.002 |
| 4 | 0.024 (0.006–0.042) | 0.009 |
| 5 | 0.028 (0.012–0.044) | 0.000 |
| 6 | 0.028 (0.012–0.044) | 0.001 |
| 7 | 0.026 (0.008–0.043) | 0.004 |
| 8 | 0.031 (0.014–0.049) | 0.000 |
| 9 | 0.039 (0.021–0.057) | 0.000 |
| 10 | 0.022 (0.006–0.038) | 0.006 |
| 11 | 0.030 (0.013–0.047) | 0.001 |
| 12 | 0.036 (0.018–0.054) | 0.000 |

MS. Multiple Sclerosis

*Adjusted for sex, age, income, education
