# Supplementary material for: Impact of surgery in patients with multiple sclerosis: a nationwide cohort study
Source: Front Neurol. 2025 Jun 26;16:1573349. doi: 10.3389/fneur.2025.1573349 (PMC12240756; doi:10.3389/fneur.2025.1573349)
Supplement: Supplementary file 3 [file Table_3.docx]

**Supplementary Table 3. Mean difference in number of MS-related diagnoses in MS-patients. comparing each month after elective surgery with pre-surgery number of MS-related diagnoses (equal to average number of MS-related diagnoses during month -12 to -7 preceding surgery).**

| **Month before/after surgery** | **Mean difference (95% CI) in number of MS-related diagnoses** | **P value** |
| --- | --- | --- |
| -6 | -0.003 (-0.019 - 0.013) | 0.726 |
| -5 | 0.005 (-0.013 - 0.022) | 0.573 |
| -4 | 0.041 (0.019 - 0.062) | 0.000 |
| -3 | 0.033 (0.013 - 0.054) | 0.001 |
| -2 | 0.042 (0.021 - 0.064) | 0.000 |
| -1 | 0.064 (0.040 - 0.089) | 0.000 |
| 1 | 0.328 (0.293 - 0.363) | 0.000 |
| 2 | 0.028 (0.004 - 0.051) | 0.020 |
| 3 | 0.003 (-0.016 - 0.021) | 0.781 |
| 4 | -0.011 (-0.030 - 0.008) | 0.249 |
| 5 | -0.010 (-0.028 - 0.008) | 0.263 |
| 6 | -0.015 (-0.033 - 0.003) | 0.098 |
| 7 | -0.014 (-0.033 - 0.005) | 0.140 |
| 8 | -0.011 (-0.029 - 0.008) | 0.264 |
| 9 | -0.001 (-0.021 - 0.0174) | 0.862 |
| 10 | -0.020 (-0.037 - -0.002) | 0.026 |
| 11 | -0.015 (-0.034 - 0.004) | 0.113 |
| 12 | -0.002 (-0.022 - 0.017) | 0.825 |

MS. Multiple Sclerosis
