# Supplementary material for: Impact of surgery in patients with multiple sclerosis: a nationwide cohort study
Source: Front Neurol. 2025 Jun 26;16:1573349. doi: 10.3389/fneur.2025.1573349 (PMC12240756; doi:10.3389/fneur.2025.1573349)
Supplement: Supplementary file 4 [file Table_4.docx]

**Supplementary Table 4. Mean difference in number of MS-related diagnoses for patients with MS compared with patients without MS before and after acute surgery*.**

| **Month before/after surgery** | **Mean difference (95% CI) in number of MS-related diagnoses** | **P value** |
| --- | --- | --- |
| -12 | 0.017 (-0.008 - 0.041) | 0.191 |
| -11 | 0.014 (-0.008- 0.036) | 0.217 |
| -10 | 0.030 (0.002 - 0.056) | 0.034 |
| -9 | 0.008 (-0.018 - 0.033) | 0.536 |
| -8 | 0.040 (0.014 - 0.067) | 0.003 |
| -7 | 0.025 (0.002 - 0.048) | 0.034 |
| -6 | 0.044 (0.013 - 0.075) | 0.005 |
| -5 | 0.027 (0.001 - 0.054) | 0.044 |
| -4 | 0.019 (-0.006 - 0.044) | 0.140 |
| -3 | 0.037 (0.007 - 0.068) | 0.018 |
| -2 | 0.031 (0.003 - 0.059) | 0.032 |
| -1 | 0.056 (0.008 - 0.105) | 0.022 |
| 1 | 0.014 (-0.056 - 0.083) | 0.697 |
| 2 | 0.026 (-0.014 - 0.065) | 0.206 |
| 3 | 0.036 (0.000 - 0.071) | 0.048 |
| 4 | 0.067 (0.029 - 0.105) | 0.001 |
| 5 | 0.030 (-0.001 - 0.060) | 0.056 |
| 6 | 0.021 (-0.007 - 0.049) | 0.141 |
| 7 | 0.028 (0.000 - 0.056) | 0.053 |
| 8 | 0.046 (0.012 - 0.081) | 0.009 |
| 9 | 0.026 (-0.002 - 0.054) | 0.067 |
| 10 | 0.039 (0.008 - 0.070) | 0.015 |
| 11 | 0.026 (-0.002 - 0.055) | 0.068 |
| 12 | 0.021 (-0.007 - 0.050) | 0.138 |

MS. Multiple Sclerosis

*Adjusted for sex, age, income, education
