# Supplementary material for: Impact of surgery in patients with multiple sclerosis: a nationwide cohort study
Source: Front Neurol. 2025 Jun 26;16:1573349. doi: 10.3389/fneur.2025.1573349 (PMC12240756; doi:10.3389/fneur.2025.1573349)
Supplement: Supplementary file 5 [file Table_5.docx]

**Supplementary Table 5. Mean difference in number of MS-related diagnoses in MS-patients. comparing each month after acute surgery with pre-surgery number of MS-related diagnoses (equal to average number of MS-related diagnoses during month -12 to -7 preceding surgery).**

| **Month before/after surgery** | **Mean difference (95% CI) in number of MS-related diagnoses** | **P value** |
| --- | --- | --- |
| -6 | 0.025 (-0.007 - 0.057) | 0.132 |
| -5 | 0.012 (-0.015 - 0.038) | 0.387 |
| -4 | 0.005 (-0.021 - 0.031) | 0.719 |
| -3 | 0.034 (0.003 - 0.066) | 0.031 |
| -2 | 0.039 (0.010 - 0.069) | 0.008 |
| -1 | 0.302 (0.255 - 0.349) | 0.000 |
| 1 | 0.722 (0.654 - 0.791) | 0.000 |
| 2 | 0.082 (0.042 - 0.121) | 0.000 |
| 3 | 0.056 (0.022 - 0.091) | 0.001 |
| 4 | 0.068 (0.030 - 0.107) | 0.001 |
| 5 | 0.022 (-0.006 - 0.052) | 0.126 |
| 6 | 0.008 (-0.020 - 0.035) | 0.585 |
| 7 | 0.016 (-0.013 - 0.045) | 0.273 |
| 8 | 0.032 (-0.004 - 0.067) | 0.078 |
| 9 | 0.009 (-0.020 - 0.038) | 0.542 |
| 10 | 0.024 (-0.009 - 0.057) | 0.156 |
| 11 | 0.009 (-0.021 - 0.038) | 0.558 |
| 12 | 0.005 (-0.024 - 0.034) | 0.717 |

MS. Multiple Sclerosis
